# Evaluation of *Pseudomonas fluorescens* for biocontrol of early blight (*Alternaria solani*) in tomato in North Wollo, Ethiopia

**DOI:** 10.1371/journal.pone.0341442

**Published:** 2026-01-23

**Authors:** Birhan Berihun Abebe, Abebe Girma Demissie, Habtie Bassie Felatie, Aderajew Adgo Tesema, Baye Wodajo, Wondye Ayaliew Shiferaw, Sualih Gobeze Hailu

**Affiliations:** 1 Department of Biotechnology, College of Natural and Computational Sciences, Woldia University, Woldia, Amhara, Ethiopia; 2 Department of Environmental and Industrial Biotechnology, Institute of Biotechnology, University of Gondar, Amhara, Ethiopia; ICAR - Indian Agricultural Research Institute, INDIA

## Abstract

Tomato (Solanum lycopersicum) is a widely used vegetable in Ethiopia, but its production is severely affected by late blight, early blight and bacterial wilt. This study aims to isolate Pseudomonas fluorescens as a bio-control agent against Alternaria solani. Biological control using Pseudomonas fluorescens offers a potential alternative to chemical fungicides. Rhizosphere soil and healthy tomato roots were sampled from three Kebeles in North Wollo, Ethiopia. P. fluorescens was isolated on Pseudomonas Isolation Agar, while A. solani isolated from infected leaves on Potato Dextrose Agar and confirmed pathogenic on tomato seedlings. Three isolates of P. fluorescens (Pfs12, Pfk13, Pfsa31) were screened in vitro using the dual culture method, and their efficacy was further tested in vivo under greenhouse conditions. Isolates Pfs12 and Pfk13 showed moderate effectiveness against the radial growth of A. solani, achieving percent growth inhibitions of 56.04% and 55.04%, respectively. The standard chemical treatment (mancozeb) resulted in a 54.84% growth inhibition. The control group (Pseudomonas fluorescens) also demonstrated a moderate growth inhibition of 57.65% against A. solani. Data were gathered regarding disease parameters. The day after transplanting, the percent disease index was significantly lower in all treated groups compared to the control (water). The isolate Pfsa31 achieved the lowest disease index of 24.733%, which was comparable to the standard chemical treatment at 28.467%. Both treatments were significantly different from the control (water) at 60.333%. The findings showed the bio-control potential of selected *P. fluorescens* isolates as effective and environmentally sustainable alternatives to synthetic fungicides for the management of early blight disease in tomato cultivation, emphasizing the importance of utilizing indigenous strains for optimal performance.

## Introduction

Tomato (*Solanum lycopersicum*) is one of the most widely cultivated and consumed horticultural crops globally. It plays a significant role in both household nutrition and income generation for farmers due to its versatility in fresh consumption and processing into products such as sauce, paste, ketchup, and juice. Tomatoes are rich in vitamins A, B, and C, and essential minerals, including potassium, phosphorus, and iron [1]. They thrive across various agro-ecological zones, typically at altitudes ranging between 700 and 2000 meters, under optimal temperatures of 21–24°C [2]. In East Africa, and particularly in Ethiopia, tomatoes are an important vegetable crop cultivated by smallholders, commercial farms, and national agricultural enterprises. The Rift Valley region, especially the Awash River Valley and lake areas, is the center of intensive tomato production using supplemental irrigation, mainly during dry seasons [3]. Despite the expansion in tomato cultivation and its growing economic significance, national average yields in Ethiopia remain below the global average and lower than those in neighboring countries [4]. According to [5], a highly significant negative correlation with EB disease resistance was observed for leaf type, mature fruit size, thickness of fruit pericarp, sepal length, petal width, and fruit shape characteristics. These tomato characteristics can be considered as favorable attributes for genetic improvement strategies through quantitative and biometrical genetics.

Several biotic and abiotic factors contribute to this yield gap, including poor agronomic practices, post-harvest handling issues, and the prevalence of destructive diseases. Among the biotic factors, fungal disease commonly occurs in tomato caused by Alternaria species. Alternaria species are a diverse group of fungi known for their role as major plant pathogens, allergens, and opportunistic pathogens in humans. includes, because of the extant diversity in pathogenicity and genetics of *A. solani* isolates, a single isolate could not be used for evaluating the resistance of potato [6]. Among the Alternaria species, *Alternaria solani* is one of the most damaging fungal pathogens causing early blight. Most studies have reported yield losses of up to 80% under severe infestation, leading to serious economic consequences for farmers [7].

To manage such diseases, many farmers rely heavily on chemical fungicides, which can offer rapid and effective control. However, their application poses several drawbacks: high costs, increased pest resistance, potential contamination of soil and water, and adverse health effects on humans and non-target organisms [8]. Additionally, the reduced availability of certain fungicides due to regulatory restrictions further limits farmers’ options, especially in low-income regions. In light of these challenges, interest has grown in biological alternatives, particularly the use of plant growth-promoting rhizobacteria (PGPR) such as *Pseudomonas fluorescens, Bacillus*, *Streptomyces*, and *Trichoderma* species [9]. PGPR are known not only for enhancing plant growth but also for their ability to suppress phytopathogens through multiple mechanisms. These include the production of antibiotics, siderophores, lytic enzymes, and the induction of systemic resistance in host plants [10]. Unlike chemical treatments, PGPR-based biocontrol is environmentally friendly, reduces chemical residue in food, and contributes to sustainable agricultural practices [11]. Among PGPR, *Pseudomonas fluorescens* has gained considerable attention due to its broad-spectrum antifungal activity. It colonizes the rhizosphere effectively and suppresses pathogens through the production of secondary metabolites such as phenazines, hydrogen cyanide (HCN), and 2, 4-diacetylphloroglucinol (DAPG), as well as through competition for nutrients and space. Several studies have confirmed the antagonistic potential of *P. fluorescens* against *A. solani,* reporting growth inhibition rates ranging from 50% to 60% in vitro [12].

Despite the promising potential demonstrated in earlier studies, there remains a critical gap in localized research. Most previous investigations were conducted outside Ethiopia, under different agro-climatic conditions and using non-native microbial strains. Very few studies have focused on identifying and evaluating indigenous *P. fluorescens* strains for their bio-control efficacy against *A. solani* under Ethiopian conditions, particularly in North Wollo, where tomato farming is widespread but suffers from high disease pressure and poor disease management practices. This region faces unique challenges, including inadequate disease control strategies, limited access to biological products, and insufficient extension services, all of which contribute to yield losses. The current research aims to isolate and screen indigenous *P. fluorescens* strains from tomato-growing areas in North Wollo, Ethiopia, and assess their in vitro antagonistic potential against *Alternaria solani* by identifying effective local isolates. This study seeks to offer sustainable, eco-friendly alternatives to chemical fungicides. Ultimately, the findings will support the development of integrated disease management strategies, contributing to improved tomato yields, enhanced farmer livelihoods, and greater food security in the region.

## Materials and methods

### Description of study area

The study was conducted at Woldia University, Biotechnology Laboratory, located in the Amhara Region, Woldia town, Ethiopia. Woldia is situated at a latitude of 11°49′ N and a longitude of 39°36′ E, at an elevation of approximately 2,112 meters above sea level. The area falls within the Weyna Dega (midland) agroecological zone and is characterized by moderate temperatures and seasonal rainfall patterns. The climate in Woldia is typically subtropical highland, with average annual temperatures ranging from 10°C to 28°C and annual rainfall between 700 mm and 1,200 mm, mainly occurring from June to September. The predominant soil types in the area are vertisols and cambisols, which support diverse agricultural production under both rainfed and irrigated conditions. Agriculture is the primary livelihood activity in Woldia and its surrounding areas, employing the majority of the population. The region is known for growing a variety of crops, including cereals (e.g., teff, sorghum, and maize), pulses (e.g., lentils and chickpeas), and horticultural crops, particularly tomatoes, onions, and peppers.

### Study design and period

The experimental research design employed a complete randomized design (CRD) to ensure systematic and unbiased results. The study took place from September 2024 to April 2025 at the Woldia University Microbial Biotechnology Laboratory, focusing on selected tomato cultivation sites in the North Wollo Zone. The research integrated both in vitro (laboratory) and in vivo (greenhouse) experiments to rigorously assess the efficacy of *Pseudomonas fluorescens* against Alternaria *solani*.

### Sample collection

Rhizosphere soil and infected tomato leaf samples were collected during September 2024 from three tomato-producing Kebeles, Sirinka, Sanka, and Kobo, located in the North Wollo Zone of the Amhara Region. These sites were selected based on a preliminary field survey assessing the prevalence and severity of early blight disease caused by *Alternaria solani*. Within each Kebele, three representative tomato farmlands showing high disease incidence were purposively selected as sampling locations. From each selected farmland, rhizosphere soil samples were collected at a depth of approximately 5 to 15 cm using sterile spatulas, and three infected tomato leaves were excised using sterilized blades. All samples were collected while wearing sterile gloves to avoid cross-contamination. In total, nine soil samples and nine leaf samples were collected from Kebele. These eighteen primary samples were then triplicated to ensure statistical validity and experimental reproducibility, resulting in fifty-four final samples (six samples per Kebele × three Kebeles × three replications), Table 1. The samples were immediately placed in sterile, labeled paper bags indicating the date and location of collection. After collection, all samples were aseptically transported in cooled containers to the Biotechnology Laboratory of Woldia University. Upon arrival, the samples were stored at 4°C until further microbiological and pathological analysis.

**Table 1:** *Pseudomonas fluorescens* treatments from study areas.

Treatments	Substrate code	Sampling sites
Trt 1	Pfs12	*Pseudomonas fluorescens* From Sirinka 1
Trt 2	Pfs21	*Pseudomonas fluorescens* From Sirinka 2
Trt 3	Pfs22	*Pseudomonas fluorescens* From Sirinka 3
Trt 4	Pfs13	*Pseudomonas fluorescens* From Kobo 1
Trt 5	Pfk21	*Pseudomonas fluorescens* From Kobo 2
Trt 6	Pfk22	*Pseudomonas fluorescens* From Kobo 3
Trt 7	Pfsa31	*Pseudomonas fluorescens* From Sanka 1
Trt 8	Pfsa32	*Pseudomonas fluorescens* From Sanka 2
Trt 9	Pfsa33	*Pseudomonas fluorescens* From Sanka 3

The experiment consisted of nine treatments and three replications. The total combination was 9*3 = 27 trials.

### Isolation of *Alternaria solani*

Tomato leaves exhibiting characteristic symptoms of early blight were collected from infected plants in the study area. Using sterile blades, the infected portions of the leaves were cut into small segments (approximately 0.5–1 cm²). These segments were surface-sterilized by immersion in 1% sodium hypochlorite solution for one minute, followed by four successive rinses with sterile distilled water to remove any residual disinfectant. The sterilized leaf tissues were then dried on sterile filter paper under aseptic conditions and placed on Petri dishes containing sterilized Potato Dextrose Agar (PDA) medium. The plates were incubated at 28°C for seven to ten days to promote the growth and sporulation of the fungal pathogen. Fungal colonies emerging from the plated tissues were carefully sub-cultured onto fresh PDA plates using a sterile inoculating needle. Single spore isolation was performed to obtain pure cultures. These pure isolates were maintained on PDA and periodically sub-cultured to preserve their viability and purity. Preliminary identification of the pathogen was based on colony morphology and growth characteristics, including texture and growth form. Further microscopic examination of the pure cultures was conducted using a light microscope at 40× magnification. Morphological characterization focused on the hyphae and conidia, particularly their shape, size, color, septation, and arrangement in chains [13].

### Preparation of A. *solani* inoculum

10 mL of sterile distilled water was poured on 14-day-old single spore PDA cultures of *Alternaria solani.* Colonies were scraped using a sterile glass slide. To remove debris, the resulting conidial suspension was sieved through a sterile muslin cloth. Using a haemocytometer, the concentration of the suspension in conidia was calculated and adjusted to 3x10^6^ spores/ml [14].

### Assessment of the pathogenicity of *A. solani*

A greenhouse experiment was conducted at Woldia University. Tomato seedlings were grown in 22 cm diameter plastic pots. Each pot was filled with a sterilized potting mixture composed of sand and sandy loam in a 2:1 ratio. The soil mixture was autoclaved at 121°C for one hour and allowed to cool for seven days before use. Five tomato seeds were sown per pot and thinned to three plants once the seedlings reached approximately 10 cm height. Pathogenicity of *Alternaria solani* was assessed following Koch’s postulates. At 40 days after sowing, healthy tomato seedlings were sprayed with a conidial suspension (3 × 10⁶ spores/mL) using a hand sprayer. Control plants were treated with sterile distilled water. To ensure optimal humidity for disease development, all inoculated and control plants were covered with transparent plastic bags for 48 hours. Environmental conditions such as temperature and humidity were closely monitored throughout the experiment. Symptoms began to appear 5–7 days post-inoculation on the lower leaves of inoculated plants. These symptoms included small, dark brown spots that gradually expanded into concentric rings characteristic of early blight. As the disease progressed, necrotic lesions enlarged, often coalescing and causing leaf chlorosis and defoliation. To confirm infection, symptomatic leaves were collected, and *A. solani* was re-isolated using standard isolation techniques. The re-isolated pathogen was cultured on PDA and compared morphologically and culturally with the original strain used for inoculation. The consistency in colony morphology and microscopic features validated the fulfillment of Koch’s postulates. Data recorded during the experiment included the number of days until symptom onset, severity of symptom expression, lesion diameter, percentage of infected leaves, plant height, and leaf chlorosis. These observations confirmed the pathogenicity of Alternaria *solani* under controlled greenhouse conditions [15].

### Isolation and purification of *Pseudomonas fluorescens*

*Pseudomonas fluorescens* was isolated from rhizosphere soil samples using the serial dilution technique. One gram of each soil sample was suspended in 9 ml of sterile distilled water and vortexed thoroughly to create a uniform suspension. Serial dilutions were prepared up to 10 ⁻ ⁷. From each dilution, 100 µL aliquots were spread on *Pseudomonas* Isolation Agar (PIA) plates using the spread plate method. The plates were incubated at 28°C for 24–48 hours to allow bacterial growth. Following incubation, distinct bacterial colonies were observed on the agar surface. Colonies with morphological characteristics typical of *Pseudomonas fluorescens*, notably their ability to fluoresce under UV light, were selected. These colonies were aseptically transferred to fresh PIA plates using a sterile inoculating needle for purification. Sub-culturing was repeated to obtain pure cultures [16]. The purified isolates were characterized based on their cultural and morphological features, including colony shape, size, pigmentation, texture, and fluorescence under ultraviolet light. Colonies that exhibited the typical greenish pigmentation and fluorescent glow were tentatively identified as *Pseudomonas fluorescens* [17].

#### Biochemical characterization of *Pseudomonas fluorescens.*

To confirm the identity of the isolates, standard biochemical tests were performed, including Gram staining, motility test [18], oxidase test, methyl red test, starch hydrolysis test, triple sugar iron test, motility test by Nepali et al. [19], catalase test [20], gelatin hydrolysis test [21], indole test [22], the fluorescein pigmentation test [23], and citrate utilization tests using Güler and Küçük [24]. Confirmed isolates were then maintained in nutrient broth supplemented with glycerol (15–20%) and stored at −20 °C for further use [25].

### In vitro evaluation of *Pseudomonas fluorescens* against Alternaria *solani*

The antagonistic potential of *Pseudomonas fluorescens* isolates against Alternaria *solani* was evaluated in vitro using the dual culture technique with three replicates per treatment. Seven-day-old cultures of A. *solani* were prepared by cutting 5 mm agar plugs using a sterile corkborer and placing them near the edge of sterile Potato Dextrose Agar (PDA) plates. Plugs were incubated for three days before inoculation of the bacterial antagonists. Isolates of *P. fluorescens*, including both newly isolated and reference strains, were spot-inoculated on the opposite side of the same plate, approximately 2 cm from the edge and equidistant from the fungal plug. Control plates with only *A. solani* served as a baseline. The plates were incubated at 28°C for five- seven days, and the radial growth of *A. solani* was measured daily from day 3 to day 13. Zones of inhibition between the bacterial colonies and fungal mycelium were recorded, and percent growth inhibition (PGI) was calculated using the formula PGI (%) = ((C − T)/ C) × 100, C- mycelia growth of the pathogen in the control T- mycelia growth of the pathogen in the dual culture plate. Based on PGI values, antagonistic activity was categorized following the scale: very low (0–25%), low (26–50%), moderate (51–75%), and high (76–100%). This in vitro screening was an essential step to identify the most promising *P. fluorescens* strains for subsequent greenhouse evaluation [26].

#### Design and set up of the pot experiments.

Microbial antagonists selected from the in vitro screening were further evaluated for their efficacy in vivo conditions using pot experiments. Certified tomato seeds of the susceptible variety Roman BF, obtained from the Sirinka Agricultural Center. Seeds were sown in sterilized nursery beds, and thirty days after sowing, healthy seedlings were transplanted into plastic pots measuring 30 cm in diameter and 30 cm in height, each filled with a sterilized potting mix composed of sand and sandy loam at a 2:1 ratio, autoclaved at 121°C for one hour. Each pot contained three rows with five plants per row, maintaining inter-row and intra-row spacing of 0.5 meters, and pots were arranged with 0.5-meter spacing between them. The experiment was arranged in a Completely Randomized Design (CRD) with three replicates per treatment. Treatments included tomato plants inoculated with *Alternaria solani* and treated with selected *Pseudomonas fluorescens* isolates (experimental treatments), plants inoculated with *A. solani* but untreated (negative control), and three plants neither inoculated nor treated (positive control). The antagonists were applied as soil drench and foliar spray at specified intervals following pathogen inoculation to assess their bio-control efficacy. The greenhouse experiment lasted for 60 days post-transplanting. Data recorded included disease incidence, severity, and index assessed from 10^th^ to 60^th^ days. Final data collection occurred at the end of the 60 days, when comprehensive assessments of disease suppression and plant growth promotion were conducted to evaluate the effectiveness of the microbial antagonists in managing early blight in tomato under controlled conditions [27].

#### Preparation of standard *Pseudomonas fluorescens.*

Standard *Pseudomonas fluorescens* was acquired from the Ethiopian Public Health Institute (EPHI) referral and reference laboratory and used as a comparison. Using a sterile micropipette, one ml of the formulation was measured and diluted as recommended for the in vitro evaluation of the bacterium against *A. solani* [28].

### Preparation of cultures filtrates from *Pseudomonas fluorescens* isolates

Culture filtrates of *Pseudomonas fluorescens* isolates were prepared using nutrient broth composed of 5 g/L peptone, 3 g/L beef or yeast extract, and 0.5 g/L sodium chloride in 1,000 mL distilled water (pH 7.0 at 25°C). Approximately 8.5 g of the medium was dissolved in 500 mL distilled water, sterilized by autoclaving at 121°C and 1 bar pressure for 15–20 minutes, and then cooled. Seven-day-old *P. fluorescens* cultures grown on *Pseudomonas* isolation agar were suspended in 10 mL sterile distilled water by flooding and scraping with sterile glass slides. This suspension was aseptically transferred into 500 mL of sterile nutrient broth in conical flasks, which were sealed with cotton wool and aluminum foil to prevent contamination. Flasks were incubated at room temperature (24 ± 2°C) on laboratory benches for 9 days. The fermentation broth was then filtered through sterile muslin cloth, and the resulting culture filtrate was collected in sterile 1,000 mL conical flasks for further use [25].

#### In vivo efficacy testing of *Pseudomonas fluorescens.*

To assess the in vivo efficacy of *Pseudomonas fluorescens,* grow the tomato plants in pots under greenhouse conditions*. P.fluorescens* suspension was prepared by culturing it in nutrient broth. Treatments were applied on a 10-day interval commencing 50 days after sowing in the greenhouse and 20 days after transplanting in the pot. One liter of culture filtrate from isolates of selected antagonists was prepared and thoroughly mixed with one ml acquawet to allow it to stick on the leaf surface. The treatments were sprayed on the leaves of tomato plants using hand sprayers. Since cases of phytotoxicity were recorded on tomato leaves for the normal strength, the culture filtrates were diluted to half strength by adding an equal volume of sterile water. One standard *Pseudomonas fluorescens*, Mancozeb or antifungal chemicals, negative control, or water, and three isolated *Pseudomonas fluorescens* were applied. A total of 6 sprays were done for the whole experiment [29]. Monitor the plants over 2–4 weeks, and signs of early blight, including leaf spots and wilting, and disease incidence were measured. Disease severity on a scale (e.g., 0–5, where 0 indicates no symptoms and 5 indicates severe symptoms [30]. In greenhouse trials, disease progression on tomato plants was monitored from the tenth to the sixteenth day after inoculation. Disease parameters such as disease incidence, severity, and disease index were recorded for each treatment group in pots, following standard procedures [31,32].


$$\text{Percent}\;\text{Disease}\;\text{Incidence}=\frac{\left(\text{Number}\;\text{of}\;\text{infected}\;\text{leaves}/\text{plants}\;\text{per}\;\text{pot}\right)}{\left(\text{Total}\;\text{number}\;\text{of}\;\text{leaves}/\text{plants}\;\text{observed}\;\text{per}\;\text{pot}\right)}\times \text{100}$$

$$\text{Percent}\;\text{Disease}\;\text{Severity}\;=\sum \frac{\left(\text{Disease}\;\text{rating}\;\times \;\text{number}\;\text{of}\;\text{plants}\;\text{scored}\;\text{for}\;\text{each}\;\text{rating}\right)}{\left(\text{Total}\;\text{number}\;\text{of}\;\text{leaves}\;\text{observed}\;\times \;\text{maximum}\;\text{rating}\;\text{scale}\right)}\times \text{100}$$

$$\;\;\text{Percent}\;\text{Disease}\;\text{Index}=\sum \frac{\left(\text{Percent}\;\text{disease}\;\text{incidence}\;+\;\text{Percent}\;\text{disease}\;\text{severity}\right)}{\left(\text{Total}\;\text{number}\;\text{of}\;\text{leaves}\;\text{per}\;\text{pot}\;\text{observed}\;\times \;\text{maximum}\;\text{rating}\;\text{scale}\right)}\times \text{1}\text{0}\text{0}\;$$

**Table:** 

Rating scale	Disease severity percent	Disease reaction
0	0	No symptom
1	1-10	Mild symptom
2	11-25	Moderate symptom
3	26-50	Symptom
4	51-75	Very Symptom
5	≥76	Totally severe

### Data analysis

The data was analyzed and interpreted using SPSS (Version 27.1). For mean comparison, Two-way ANOVA was used to determine statistical significance among treatments and fishers protected least significance difference (LSD) test at 5% significance level. Finally, correlation analysis was used under in vivo conditions to link the relationship between disease parameters (Incidence, severity and Index).

## Results

### Isolation and morphological characterization of isolated *Alternaria solani*

**Fig 1:**
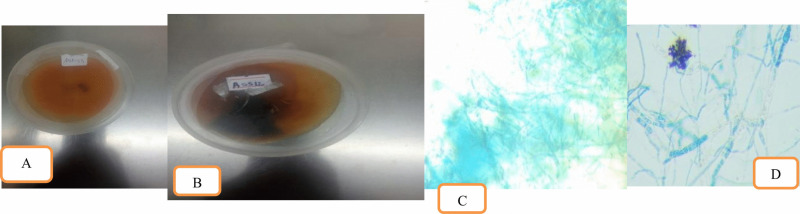
Cultural and morphological characteristics of isolated *Alternaria solani.* (A) *Alternaria solani* seven- day old colony (obverse), (B) *Alternaria solani* ten- day old colony (reverse), (C) *Alternaria solani* hyphae (x40), (D) *Alternaria solani* conidia (x40).

Pure colonies of Alternaria *solani* that were grown on Potato Dextrose Agar (PDA) exhibited distinct characteristics for identification. The morphological and microscopic characterization of *Alternaria solani* isolates revealed distinct variations across several features. Colony coloration ranged from white (Ass11, Assa12, Ask12, Ask13) to dark brown (Ass12, Ass13, Assa13, Ask11), grey (Ass13), and blackish hues (Assa11). Colony textures varied from cottony (Ass11, Assa12, Ask11, Ask12, and Ask13) to fluffy (Ass12, Ass13, Assa11, and Assa13). Growth patterns were predominantly fast and smooth, with isolates showing either regular (Ass11, Ass13, Assa12, Ask11) or irregular growth (Ass12, Assa11, Ask12). The reverse sides of the colonies exhibited primarily dark brown (Ass11, Ass13, Assa13, Ask12) or white pigmentation (Ass12, Assa12, Ask11, Ask13), with one isolate (Assa11) displaying a yellow reverse. Colony shapes were mainly circular and raised (Ass13, Assa11, Assa12, Assa13, Ask11, Ask12), with some exhibiting hairy margins (Ass11, Ass12, Assa13, Ask13). Colony sizes varied significantly, ranging from 36.9 µm (Ass13) to 93.2 µm (Ask13). Microscopic analysis showed that the hyphae were mostly branched filaments (Ass12, Ass13, Assa13, Ask11, Ask12, Ask13), with some isolates forming septate hyphae with chains (Ass11) or bearing conidia with beaks (Assa11, Assa12). Septation patterns included horizontal septation ranging from 2 to 7 and vertical septation from 1 to 4, providing a comprehensive profile of the morphological and microscopic diversity among the examined A. *solani* isolates. A detailed morphological analysis had been presented in Fig 1 and Table 2.

**Table 2:** Cultural, morphological and microscopic characteristics of isolated Alternaria *solani.*

Isolate Code	Color	Texture	Growth Form	Reverse Side	Shape	Size (µm)	Hyphae	Septation	
								Horizontal	Vertical
Ass11	White	Cottony	Fast, Smooth, regular	Dark brown	hairy margin	78.4	Septate hyphae, septate chain forming	3-5	1-3
Ass12	Dark brown	Fluffy	Weak, Smooth, irregular	White	hairy margin	42.5	branched filament	3-6	2-4
Ass13	Grey	Fluffy	Fast, Smooth, regular	Dark brown	Circular, raised colonies	36.9	branched filament	3-5	1-2
Assa11	Blackish	Fluffy	Fast, Smooth, irregular	Yellow	Circular, raised colonies	38.7	Septate, Beaks – like structure	2-6	1-3
Assa12	White	Cottony	Fast, Smooth, regular	White	Circular, raised colonies	91.5	Septate, Beaks on conidia	3-7	1-3
Assa13	Dark brown	Fluffy	Fast, Flat growth circular	Dark brown	hairy margin	75.4	branched filament	4-7	1-2
Ask11	Dark brown	Cottony	Medium, Smooth, regular	White	Circular, raised colonies	70.3	branched filament	4-7	1-2
Ask12	White	Cottony	Fast, Smooth, irregular	Dark brown	Circular, raised colonies	92.3	branched filament	2-5	1-2
Ask13	White	Cottony	Fast	White	hairy margin	93.2	branched filament	2-6	1-3

### Pathogenicity of Alternaria *solani*

**Fig 2:**
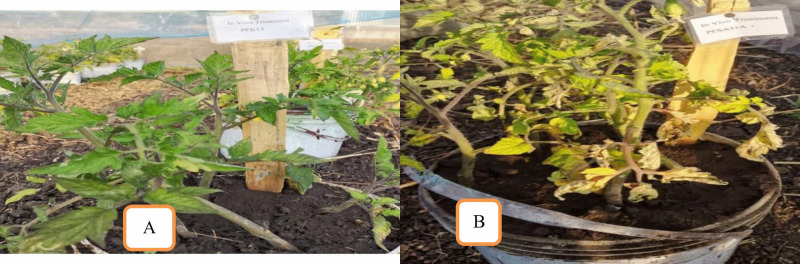
Early blight symptoms on an infected tomato leaf and a healthy tomato leaf under greenhouse. (A) Healthy tomato leaf from greenhouse pot, (B) early blight symptoms on infected tomato leaf from greenhouse pot.

In this study, 20 days old tomato seedlings were inoculated with a conidial suspension of *Alternaria solani.* They developed characteristic early blight symptoms 15 days after inoculation. Affected foliage displayed dark brown, oval to angular lesions measuring 2–7 mm in diameter, exhibiting distinct concentric rings typical of A*. solani* infection. Lesions gradually expanded, leading to complete blighting of the infected leaves. To satisfy Koch’s postulates, the pathogen was re-isolated from symptomatic leaf tissues using standard tissue isolation techniques on Potato Dextrose Agar (PDA). The resulting single-spore colonies exhibited cultural and morphological characteristics identical to those of the original isolate. Microscopic examination confirmed that the hyphal and conidial features of the re-isolated fungus were consistent with *A. solani*, thereby confirming the identity of the pathogen, Fig 2. Therefore, this re-isolated pathogen was the causal agent.

### Morphological characterization of isolated Pseudomonas *fluorescens*

**Fig 3:**
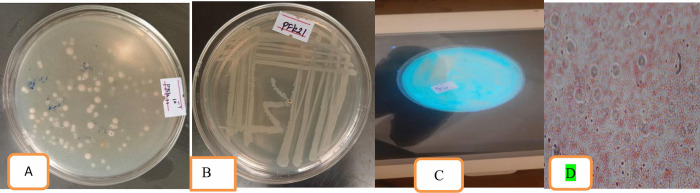
Cultural and morphological characteristics of isolated *Pseudomonas fluorescens.* (A) *Pseudomonas fluorescens* colonies, (B) *Pseudomonas fluorescens* subculturing, (C) Isolated *Pseudomonas fluorescens* under ultraviolet light, (D) Microscopic observation of *Pseudomonas fluorescens*.

The bacteria Pseudomonas *fluorescens* were isolated from rhizosphere soil using Pseudomonas isolation agar. One gram of rhizosphere soil was collected and serially diluted up to 10^−7^. From this dilution, 27 distinct bacterial colonies were isolated. Among 27 *Pseudomonas fluorescens* isolates, only nine were morphologically characterized based on features such as colony shape, cell shape, color, elevation, surface texture, pigmentation, and Gram reaction. In terms of surface coloration, most isolates displayed a greenish tint (Pfs12, Pfsa31, Pfsa32, Pfsa33, and Pfk13), while others showed a blue-green tint (Pfs21, Pfs22, Pfk21, Pfk22). The reverse side of the colonies was predominantly yellowish-green in Pfs12, Pfs21, Pfs22, Pfsa31, Pfsa32, and Pfsa33; whereas Pfk13, Pfk21, and Pfk22 exhibited off-white reverse. Colony textures ranged from smooth (Pfs12, Pfsa31, Pfsa32, Pfsa33, Pfk21, Pfk22) to glossy (Pfs21, Pfs22, Pfk13). The colony margins were generally rounded, except for Pfk13, which displayed an irregular margin. All isolates were rod-shaped, with colony sizes ranging from 1 to 3 mm. Cellular arrangement of most isolates formed small clusters (Pfs12, Pfs21, Pfs22, Pfk21, Pfk22), while Pfsa31, Pfsa32, Pfsa33, and Pfk13 appeared as single cells. Colony elevation was predominantly convex, except in Pfk21 and Pfk22, where a flat elevation was observed. These findings provide a comprehensive morphological profile of the isolated *P. fluorescens* strains, as illustrated in Fig 3 and Table 3.

**Table 3:** Cultural and morphological characterization of isolated *Pseudomonas fluorescens.*

Sampling Code	Surface Color	Reverse color	Texture	Margin	Shape	Size	Arrangement	Elevation
**Negative Control**								
***P. fluorescens***	Greenish	Yellowish green	Smooth	Round	Rod	1-3 mm	small cluster	Convex
**Pfs12**	Greenish	Yellowish green	Smooth	Round	Rod	1-3 mm	small cluster	Convex
**Pfs21**	Blue Green	Yellowish green	Glossy	Round	Rod	1-3 mm	small cluster	Convex
**Pfs22**	Blue Green	Yellowish green	Glossy	Round	Rod	1-3 mm	small cluster	Convex
**Pfsa31**	Greenish	Yellowish green	Smooth	Round	Rod	1-3 mm	Single cell	Convex
**Pfsa32**	Greenish	Yellowish green	Smooth	Round	Rod	1-3 mm	Single cell	Convex
**Pfsa33**	Greenish	Yellowish green	Smooth	Round	Rod	1-3 mm	Single cell	Convex
**Pfk13**	Greenish	Off white	Glossy	Irregular	Rod	1-3 mm	Single cell	Convex
**Pfk21**	Blue Green	Off white	Smooth	Round	Rod	1-3 mm	small cluster	Flat
**Pfk22**	Blue Green	Off white	Smooth	Round	Rod	1-3 mm	small cluster	Flat

### Biochemical characterization of isolated *Pseudomonas fluorescens*

Out of the 27 *Pseudomonas fluorescens* isolates obtained, nine representative isolates were selected for further detailed biochemical characterization and analysis. All nine isolates tested were positive for citrate utilization, catalase activity, oxidase activity, motility, triple sugar iron (TSI) reactions, fluorescent pigment production, and gelatin liquefaction- traits that are characteristic of *P. fluorescens* and support their species-level identification. In contrast, all isolates were negative for starch hydrolysis and indole production. They exhibited Gram-negative staining, confirming their Gram-negative cell wall structure characterized by a thin peptidoglycan layer and an outer membrane, which indicates specific metabolic limitations. Catalase and oxidase positivity confirmed the ability of the isolates to break down hydrogen peroxide and the presence of cytochrome-c oxidase, respectively, hallmark features of *Pseudomonas* species. Motility tests revealed that all isolates were motile and possessed flagella, suggesting enhanced adaptability to diverse environments. The starch hydrolysis and indole tests confirmed the absence of amylase and the inability to convert tryptophan to indole, consistent with the known biochemical profile of *P. fluorescens*. TSI agar tests showed that most isolates fermented glucose without producing hydrogen sulfide (H₂S) and did not ferment lactose or sucrose. However, isolates Pfsa31, Pfsa32, Pfk21, and Pfk22 did not ferment glucose, suggesting possible strain-specific variations or experimental inconsistencies. All isolates were gelatinase-positive, and citrate utilization confirmed their ability to use citrate as a sole carbon source. Additionally, the methyl red test was positive in all isolates, indicating stable acid production during glucose metabolism. The production of fluorescent pigments under UV light further confirmed the identity of the isolates *as P. fluorescens*. A detailed summary of these results is presented in Table 4 and a supportive image S1 Fig in S1 Fig.

**Table 4:** Biochemical characteristics of isolated *Pseudomonas fluorescens.*

Isolate Code	Gram Staining	Catalase test	Oxidase Test	Motility Test	Starch hydrolysis test	Indole test	Gelatin liquefaction Test	Methyl red test	Citrate Utilization Test	*fluorescens* Pigmentation test
Negative Control										
*P. fluorescens*	**–**	_**+**_	_**+**_	_**+**_	**–**	**–**	_**+**_	_**+**_	_**+**_	_**+**_
Pfs12	**–**	_**+**_	_**+**_	_**+**_	**–**	**–**	_**+**_	_**+**_	_**+**_	_**+**_
Pfs21	**–**	_**+**_	_**+**_	_**+**_	**–**	**–**	_**+**_	_**+**_	_**+**_	_**+**_
Pfs22	**–**	_**+**_	_**+**_	_**+**_	**–**	**–**	_**+**_	_**+**_	_**+**_	_**+**_
Pfsa31	**–**	_**+**_	_**+**_	_**+**_	**–**	**–**	_**+**_	_**+**_	_**+**_	_**+**_
Pfsa32	**–**	_**+**_	_**+**_	_**+**_	**–**	**–**	_**+**_	_**+**_	_**+**_	_**+**_
Pfsa33	**–**	_**+**_	_**+**_	_**+**_	**–**	**–**	_**+**_	_**+**_	_**+**_	_**+**_
Pfk13	**–**	_**+**_	_**+**_	_**+**_	**–**	**–**	_**+**_	_**+**_	_**+**_	_**+**_
Pfk21	**–**	_**+**_	_**+**_	_**+**_	**–**	**–**	_**+**_	_**+**_	_**+**_	_**+**_
Pfk22	**–**	_**+**_	_**+**_	_**+**_	**–**	**–**	_**+**_	_**+**_	_**+**_	_**+**_

Key: + : indicates positive, -: indicates negative.

### In vitro evaluation of isolated *Pseudomonas fluorescens* against Alternaria *solani*

*Pseudomonas fluorescens* isolates coded Pfs12, Pfs21, Pfs22, Pfk13, Pfk21, Pfk22, Pfsa31, Pfsa32, and Pfsa33 were selected based on preliminary experiments, along with a standard *P. fluorescens* strain obtained from the Ethiopian Public Health Institute (EPHI). The anti-fungal chemicals (mancozeb) were evaluated for their in vitro antagonism activity against A. *solani*. Each treatment was replicated three times. The experimental design was a Complete Randomized Design (CRD) in triplicate. Data were taken on the growth of the pathogen. The radial growth of the pathogen’s colonies (measured in centimeters) was recorded daily, beginning on the third day after the bio-control agents (BCAs) were introduced, until the thirteenth day, when no further growth was observed in the control plates. This experimental result showed that the selected *P. fluorescens* isolates, anti-fungal chemicals (mancozeb), and the standard strain significantly inhibited the radial growth of *A. solani* from the third day onward, with suppression continuing until no additional colony expansion was detected in the control plates.

**Fig 4:**
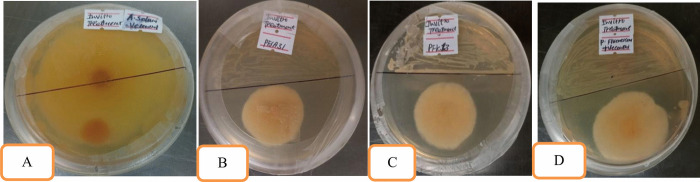
Mean diameter of radial growth of pathogen in the presence of isolated *Pseudomonas fluorescens.*

**Fig 5:**
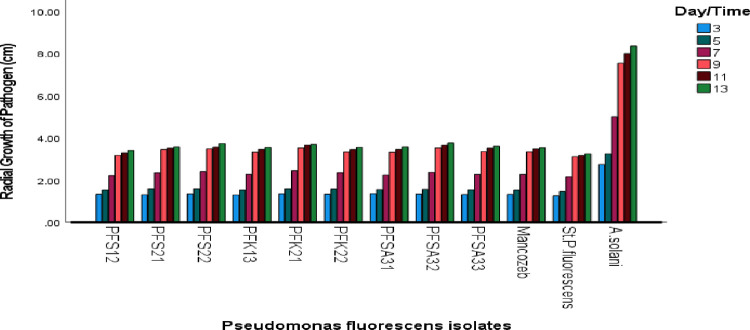
Mean diameter of radial growth of pathogen in the presence of isolated *Pseudomonas fluorescens.*

Nine *P. fluorescens* isolates, standard check (mancozeb), and control (*Pseudomonas*) strain were screened for their inhibitory effect on the radial growth of *A. solani* using the dual culture technique. All isolates demonstrated moderate growth inhibition against *A. solani*. The pathogen's growth in dual culture plates continued until it reached the leading edge of the antagonists. In the in vitro antagonism activity of the radial growth of the pathogen, radial growth of treatment and percent growth inhibition did not significantly (at p > 0.05) differ among treatments on the day after transplanting. Significant (p ≤ 0.05) increases were recorded for the radial growth of the pathogen, radial growth of treatments, and percent growth inhibition in all the treatments with time. Comparison of means was done using Fisher’s protected LSD test (at p ≤ 0.05) using SPSS version 27.1, and the mean interaction effect was performed using Statstix 10. Among the different isolates of *P. fluorescens* and anti-fungal chemicals (mancozeb), the standard strain exhibited the highest suppression, recording the least pathogen colony diameter (2.40 cm), followed by PFS12 (2.49 cm), PFK13 (2.57 cm), and Pfsa31 (2.58 cm), respectively. The standard *P. fluorescens* strain exhibited the highest percent growth inhibition (57.65%), followed by PFS12 (56.04%), PFK13 (55.04%), and PFK21 showed the lowest antagonistic effect (52.91%). Isolates like PFS12 and PFK13 displayed moderate inhibitory effects, while the standard *P. fluorescens* strain demonstrated a stronger antagonistic potential. In this study, no zones of growth inhibition were observed between the colonies of *P. fluorescens* and those of *A. solani.* The detailed in vitro antagonism activity of *P. fluorescens* isolates, control and standard check was summarized in Figs 4 and 5, Tables 5 and 6.

**Table 5:** Antagonistic activity of isolated *Pseudomonas fluorescens*, standard check (Mancozeb) and control (standard P.*fluorescens*) against Alternaria *solani* with treatments.

Experimental site	Treatments	Radial growth of pathogens(cm)	Radial growth of treatments(cm)	Inhibition (%)
	*A. solani*	5.81a	.00e	.00g
	Pfs12	2.49e	3.32ab	56.04b
	Pfs21	2.63c	3.18 cd	53.98e
	Pfs22	2.68b	3.12c	53.07f
	Pfk13	2.57d	3.24bc	55.04c
	Pfk21	2.71b	3.10d	52.91f
	Pfk22	2.60 cd	3.21a	54.23de
In vitro	Pfsa31	2.58d	3.23c	54.65cde
	Pfsa32	2.70b	3.11d	53.05f
	Pfsa33	2.60 cd	3.21c	54.61cde
	Mancozen	2.58d	3.23c	54.84 cd
	St.P*.fluorescens*	2.40f	3.41a	57.65a
	Grand Mean	2.8607	2.9465	50.006
	C.V (%)	1.74	4.46	2.31
	LSD (p ≤ 0.05)	0.0327	0.0866	0.7619

Means followed by the same letter (s) in the same column did not significantly differ (at 5%). *Pseudomonas fluorescens* isolates: Pfs12 *(Pseudomonas fluorecsens* from Sirinka), Pfs21(*Pseudomonas fluorecsens* from Sirinka), Pfs22 (*Pseudomonas fluorescens* from Sirinka), Pfk13 *(Pseudomonas fluorescens* from Kobo), Pfk21 (Pseudomonas *fluorecsens* from Kobo), Pfk22 (*Pseudomonas fluorescens* from Kobo), Pfsa31 (*Pseudomonas fluorescens* from Sanka), Pfsa32 (*Pseudomonas fluorescens* from Sanka) and Pfsa33 (*Pseudomonas fluorescens* from Sanka), St*.*.*P. fluorescens*: Positive control, mancozeb: standard check, A *A. solani*: Negative control, mean: grand mean, C.V (%): coefficient of variation, LSD: least significant difference.

**Table 6:** Antagonistic activity of isolated *Pseudomonas fluorescens*, standard check (Mancozeb) and control (standard *P. fluorescens*) against *Alternaria solani* with days.

Experimental Site	Day	Mean of RGP (cm)	Mean of RGT (cm)	Mean of PGI (%)
	13	3.9619a	4.3781a	52.487a
	11	3.8475b	4.1425b	51.840b
	9	3.7039c	3.8294c	50.787c
	7	2.5292d	2.4708d	49.354d
In vitro (In laboratory)	5	1.6858e	1.5608e	48.038e
	3	1.4358f	1.2975f	47.530e
	Grand Mean	2.8607	2.9465	50.006
	C.V (%)	1.74	4.46	2.31
	LSD (p ≤ 0.05)	0.0231	0.0612	0.5388

**Fig 6:**
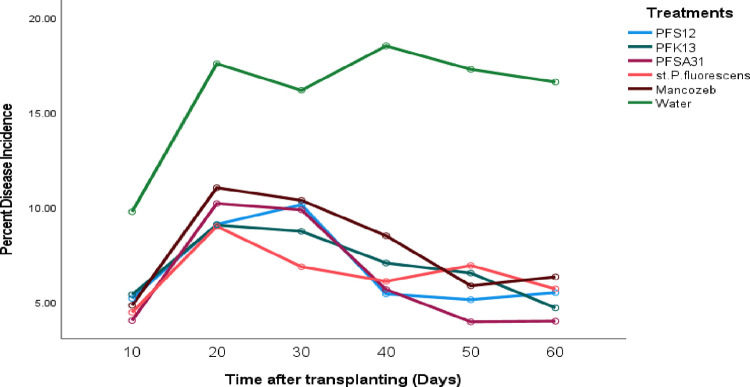
Effect of bio-control agents on the percent disease incidence for the tomato early blight disease progress curve of marginal mean at the greenhouse.

Means of RGP and RGT were significantly different from one another (at 5%) but means of PGI did not significantly different from one another (at 5%). Day: day after plating bacterial antagonists, RGP: radial growth of pathogen, RGT: radial growth of treatment, PGI: percent growth inhibition, Mean: grand mean, C.V (%): coefficient of variation, LSD: least significance difference Fig 6.

### In vivo efficacy testing of isolated *Pseudomonas fluorescens* in the managing of tomato early blight

In this study, six promising BCAs with notable inhibitory effects on the in vitro growth of *Alternaria solani* were selected for evaluation. Among these, four treatments were chosen for greenhouse trials to assess their effectiveness in controlling early blight pathogen in tomatoes. The BCAs included three isolates of *Pseudomonas fluorescens* (Pfs12, Pfk13, and Pfsa31) and one strain of *Pseudomonas fluorescens*. Water served as the control treatment, while mancozeb was used as the standard chemical check. Each treatment was replicated three times. The experiment followed by Complete Randomized Design with three replications. Mean comparisons were performed using Fisher’s protected LSD test (at p ≤ 0.05) with SPSS version 27.1 and mean interaction effect was performed using Statstix 10. Results showed that percent disease incidence was significantly lower in all BCA treatments compared to the control (water). The disease incidence observed with mancozeb was comparable to that of BCAs. Similarly, percent disease severity and the percent disease index were significantly lower in all treatments relative to the control (water). Disease incidence ranged from 6.26% to 7.79% across all treatments, while the control treatment (water) had a significantly higher incidence of 15.94%. Disease severity ranged from 5.67% to 6.75% for all treatments, whereas the control treatment recorded a significantly higher severity of 14.22%. The percent disease index ranged from 24.29% to 28.47% for all treatments, while the control treatment had a significantly higher disease index of 60.33%. The findings suggest that BCAs are effective in managing early blight in tomatoes under greenhouse conditions, reducing growth with the disease.

### Percent disease incidence for early blight in tomato plants treated with the various antagonists

In the greenhouse experiment, the percent disease incidence for tomato early blight did not significantly vary (at p > 0.05) among treatments starting from the day after transplanting. Over time, a significant (p ≤ 0.05) increase in disease incidence was observed in all treatments. However, all treatments significantly (p ≤ 0.05) reduced the percent disease incidence compared to the control treatment (water) throughout the experiment, Table 7. Specifically, on the 10^th^, 40^th^, 50^th^, and 60^th^ days after transplanting, the standard check (mancozeb) and *P. fluorescens* isolates coded Pfs12, Pfk13, and Pfsa31 significantly (p ≤ 0.05) lowered the percent disease incidence compared to the control (water). The percent disease incidence ranged from 6.255% to 7.7889% for all treatments, while the control (water) treatment recorded a significantly moderate disease incidence of 15.944%. Significant differences (p ≤ 0.05) in percent disease incidence were observed between all treatments and the control (water) from the 10^th^ to the 60^th^ day after transplanting (**Table 6**). This study demonstrated the efficacy of selected microbial antagonists in reducing early blight on tomato plants under greenhouse conditions. The application of these antagonists significantly protected tomato leaves from *A. solani* infection and prevented the pathogen from spreading across leaf surfaces. By mitigating the impact of *A. solani,* the antagonists helped maintain the photosynthesis process, thereby supporting overall plant health and productivity. Detailed data on the percent disease incidence for *P. fluorescens* isolates, the control, and the standard check are summarized in Fig 6 and Table 7.

**Table 7:** Percent disease incidence for early blight in tomato plants treated with the various antagonists with treatments and days.

Experimental site	Treatments	Mean of PDI	Day	Mean of PDI
	PFS12	6.7167 cd	60	7.106d
	PFK13	6.8833c	50	7.583d
	PFSA31	6.2556d	40	8.511c
In vivo (Greenhouse)	St.P*.fluorescens*	6.4778 cd	30	10.322b
	Mancozeb	7.7889b	20	10.961a
	Water	15.944a	10	5.583e
	Grand Mean	8.3444		8.3444
	C.V (%)	10.41		10.41
	LSD (p ≤ 0.05)	0.5777		0.5777

Means followed by the same letter (s) in the same column and experimental site did not significantly different (at 5%) for both treatment and day. LSD: Least significant difference, % CV: Percent of coefficient of variation*, Pseudomonas fluorescens* isolates: PFS12, PFK13 and PFSA31, St. *P. fluorescens:* Standard *Pseudomonas fluorescens*, Mancozeb: Synthetic fungicide, water: control, PDI: percent disease index.

### Percent disease severity for early blight in tomato plants treated with the various antagonists

**Fig 7:**
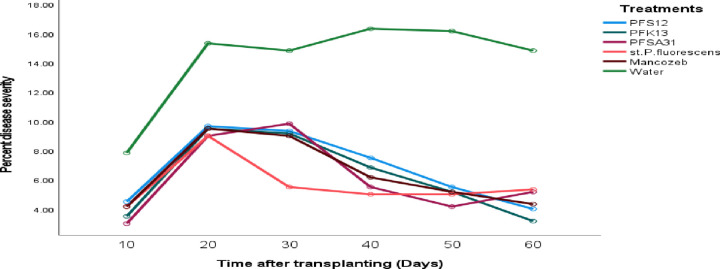
Effect of biocontrol agents on percent disease severity for tomato early blight disease progress curve of marginal mean at greenhouse.

In the greenhouse, the percent disease severity for tomato early blight did not significantly varied (at p > 0.05) among treatments starting from the day after transplanting. Over time, a significant (p ≤ 0.05) increase in disease severity was observed in all treatments. However, all treatments significantly (p ≤ 0.05) reduced the percent disease severity compared to the control (water) treatment as the experiment progressed. The percent disease severity ranged between 5.667% and 6.750% for all treatments; while the control (water) treatment recorded a significantly moderate disease severity of 14.222%. The percent disease severity was somewhat lower in pots treated with mancozeb compared to those treated with the various microbial antagonists, though the differences were not substantial. Detailed data on the percent disease severity for *P. fluorescens* isolates, the control, and the standard check are summarized in Fig 7 and Table 8.

**Table 8:** Percent disease severity for early blight in tomato plants treated with the various antagonists with treatments and days.

Experimental site	Treatments	Mean of PDS	Day	Means of PDS
	Pfs12	6.750b	60	6.139e
	Pfk13	6.222bc	50	6.861d
	Pfsa31	6.111 cd	40	7.889c
In vivo (Greenhouse)	St. P*. fluorescens*	5.667d	30	9.611b
	Mancozeb	6.389bc	20	10.333a
	Water	14.222a	10	4.528f
	Grand Mean	7.5602		7.5602
	C.V (%)	11.05		11.05
	LSD (p ≤ 0.05)	0.5552		0.5552

Means followed by the same letter (s) in the same column and experimental site did not significantly differ (at 5%) for treatment but means were significantly different from one another (at 5%) for day. LSD: Least significant difference, % CV: Percent of coefficient of variation, *Pseudomonas fluorescens* isolates: PFS12, PFK13, and PFSA31, St. *P*. *fluorescens*: Standard *Pseudomonas fluorescens*, Mancozeb: Synthetic fungicide, water: control, PDS: percent disease severity.

### Percent disease index for early blight in tomato plants treated with the various antagonists

**Fig 8:**
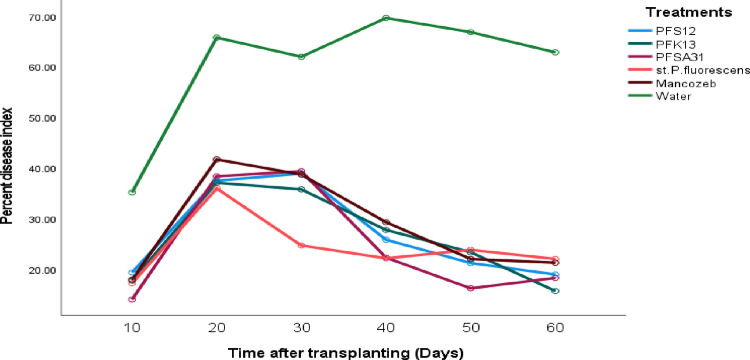
Effects of bio-control agents on the percent disease index for the tomato early blight disease progress curve of the marginal mean at the greenhouse.

In the greenhouse, the percent disease index for tomato early blight did not significantly vary (at p > 0.05) among treatments starting from the day after transplanting. Over time, significant (p ≤ 0.05) increases in the percent disease index were observed in all treatments. However, all treatments significantly (p ≤ 0.05) reduced the percent disease index compared to the control (water) treatment as the experiment progressed. The percent disease index ranged between 24.289% and 28.467% for all treatments, while the control treatment recorded a significantly higher disease index of 60.333%. The effects of mancozeb in reducing the percent disease index were significantly (p ≤ 0.05) different from those of the various microbial antagonists, demonstrating its superior efficacy. Detailed data on the percent disease index for the treatments, including *P. fluorescens* isolates, the control, and the standard check, were summarized in Fig 8 and Table 9. These findings underscore the effectiveness of the tested treatments in minimizing disease index under greenhouse conditions.

**Table 9:** Percent disease index severity for early blight in tomato plants treated with the various antagonists, with treatments and days.

Experimental site	Treatment	PDIx	Day	Mean of PDIx
	Pfs12	26.933c	60	26.489e
	Pfk13	26.211 cd	50	28.889d
	Pfsa31	24.733de	40	32.800c
In vivo (Greenhouse)	St.P*.fluorescens*	24.289e	30	39.867b
	Mancozeb	28.467b	20	42.700a
	Water	60.333a	10	20.222f
	Grand Mean	31.828		31.828
	C.V (%)	7.08		7.08
	LSD (p ≤ 0.05)	1.4974		1.4974

Means followed by the same letter (s) in the same column and experimental site did not significantly differ (at 5%) for treatments, but means were significantly different from one another (at 5%) for day. LSD: Least significant difference, % CV: Percent of coefficient of variation, Pfs12, Pfk13 and Pfsa31, St. *P. fluorescens*: Standard *Pseudomonas fluorescens*, Mancozeb: Synthetic fungicide, water: control, PDIx: percent disease index.

### Correlations between tomato early blight disease growth parameters

The in vitro assays demonstrated that *Pseudomonas fluorescens* isolates, particularly Pfs12, Pfk13, and Pfsa31, produced larger zones of inhibition against *A. solani*, indicating strong antagonistic potential. These results translated effectively into greenhouse trials, where the same isolates significantly reduced disease incidence, severity, and index in tomato plants. This correlation suggests that the antimicrobial compounds produced by these isolates in vitro likely play a vital role in suppressing pathogen growth in tomato. In the greenhouse, the percent disease incidence, percent disease severity, and percent disease index for tomato early blight did not significantly vary (at p > 0.05) among treatments starting from the day after transplanting. Correlations were recorded between different disease parameters. After a time at the greenhouse, the percent disease incidence, percent disease severity, and percent disease index were significantly varied (at p ≤ 0.05) and (at p ≤ 0.01), Table 10.

**Table 10:** Correlations between tomato early blight disease growth parameters.

	Percent Disease Incidence	Percent Disease Severity	Percent Disease Index
Percent Disease Incidence	1	.935^**^	.985^**^
		.000	.000
	108	108	108
Percent Disease Severity	.935^**^	1	.982^**^
	.000		.000
	108	108	108
Percent Disease Index	.985^**^	.982^**^	1
	.000	.000	
	108	108	108

**Key:** The table indicates correlation between tomato early blight disease parameters, **: highly Significant (at p ≤ 0.01) and *: Significant (at p ≤ 0.05).

## Discussion

The successful isolation of *Alternaria solani* from infected tomato leaves provided essential morphological evidence consistent with established descriptions of this pathogen. The colonies displayed characteristic pigmentation, dark on the reverse side and greyish on the front, which is a unique characteristic of *A*. *solani*. Microscopic examination revealed septate, branched hyphae that darkened over time. Conidiophores were short, septate, and brownish, while the conidia were large, brownish, and occurred singly or in pairs. The presence of 2 to 7 horizontal septa and 1 to 4 vertical septa further supported the identification of *A. solani*. These morphological characteristics align with previous reports and reflect the variation among *A. solani* strains, which may influence their pathogenicity and response to management strategies [13,33,34]. A pathogenicity test conducted in a greenhouse confirmed the disease-causing ability of the *A. solani* isolates. Fifteen days after inoculation, tomato plants exhibited early blight symptoms, including dark brown, oval to angular lesions (2–7 mm) with concentric rings, which expanded over time and led to complete leaf blighting. The pathogen was successfully re-isolated from the infected leaves, confirming Koch’s postulates. These symptoms are consistent with those reported in previous studies [35–37].

*Pseudomonas fluorescens* was isolated from rhizosphere soil using Pseudomonas Isolation Agar. From serial dilutions up to 10 ⁻ ⁷. A total of 27 bacterial colonies were obtained, of which nine were selected for further morphological characterization based on colony shape, cell shape, color, surface texture, pigmentation, elevation, and Gram reaction. These features were consistent with descriptions of *P. fluorescens* reported in earlier studies [23,38,39]. Biochemical characterization of the nine isolates confirmed their identity as *P. fluorescens*. All isolates tested positive for citrate utilization, catalase activity, oxidase activity, motility, triple sugar iron reactions, fluorescent pigment production, and gelatin liquefaction, and negative for starch hydrolysis, Gram staining, and indole production. This biochemical profile supports their classification as *P*. *fluorescens* and highlights their metabolic capabilities relevant to bio-control functions [40,41]. In vitro testing of the antagonistic potential of *P. fluorescens* against A. *solani* was carried out using the dual culture technique. Radial growth measurements were taken from day 3 to day 13 following inoculation. All *P. fluorescens* treatments, along with the standard strain and the fungicide mancozeb, significantly inhibited the growth of *A. solani* compared to the control. The most effective isolates were Pfs12, Pfk13, and Pfsa31, with radial growth measurements of 2.49 cm, 2.57 cm, and 2.58 cm, respectively, compared to 2.40 cm for the standard strain. Percent growth inhibition was highest for the standard strain (57.65%), followed by Pfs12 (56.04%), Pfk13 (55.04%), and Pfsa31 (54.65%). The lowest inhibition (52.90%) was recorded for Pfk21. These findings are consistent with those of [42], who reported growth inhibition percentages between 47% and 60%.

These results confirm the effectiveness of local *P. fluorescens* isolates and support their use as sustainable alternatives to chemical fungicides. Similar outcomes were reported by [32,26], who demonstrated the efficacy of microbial bio-control agents, including *P. fluorescens*, in managing early blight. However, [32] also noted that the performance of some isolates was comparable to the standard strain, indicating that efficacy may vary depending on the isolate.

Clear inhibition zones between Pseudomonas colonies and A. *solani* suggest the production of secondary metabolites responsible for antifungal activity. This observation aligns with previous studies that documented the antifungal potential of *P. fluorescens* secondary metabolites [43]. Additionally, *P. fluorescens* demonstrated the ability to overgrow and mycoparasitize A. *solani*, further supporting its bio-control potential.

In vivo efficacy testing under greenhouse conditions evaluated the effectiveness of microbial treatments, the standard *P. fluorescens* strain, and mancozeb in managing early blight. Initially, there were no statistically significant differences (p > 0.05) among treatments in terms of disease incidence, severity, and index. However, significant differences (p ≤ 0.05) emerged from day 10 to day 60 post-transplantation. All treatments significantly reduced disease progression compared to the untreated control. Disease incidence in treated plants ranged from 6.25% to 7.79%, while the untreated control showed 15.94%. Disease severity ranged from 5.67% to 6.75% in treated pots, compared to 14.22% in the control. Although slightly less effective than the fungicide and standard strain, *P. fluorescens* isolates still provided substantial disease suppression. These results align with findings from [32,44], who highlighted the effectiveness of *Trichoderma spp.*, Bacillus *spp.*, and *P. fluorescens* in managing early blight.

Similarly, the disease index was significantly reduced (p ≤ 0.05) in treated plants, ranging from 24.29% to 28.47%, compared to 60.33% in the control. The superior efficacy of *P. fluorescens* isolates, compared to chemical and standard treatments, underscores their potential as bio-control agents [45]. While chemical fungicides achieved slightly higher reductions (85%), the microbial treatments still provided significant disease control, supporting their integration into sustainable management strategies. According to the current study, disease severity ranges from 5.667 to 14.22 at the transplanting stage (within 10 days). This result aligns with the study by [46], who record disease severity on different tomato genotypes ranging from 5 (H.a.s 2274, US) to 18.5 (Ameera RZ, Netherlands). However, as the stage of the tomato plants increases from 10 to 60, the disease severity becomes reduced, which is due to the reason that the bio-control method of different bacteria becomes effective over such *Alternaria solani* disease. The reduction in disease incidence, severity, and index suggests that microbial antagonists act through various mechanisms, including competition for nutrients, production of antimicrobial compounds, and the induction of systemic resistance. These findings support the use of *P. fluorescens* as a biological alternative to synthetic fungicides.

Overall, the results highlight the potential of *P. fluorescens* isolates, particularly Pfs12, Pfk13, and Pfsa31, as effective and environmentally friendly alternatives to chemical fungicides. In vitro and in vivo experiments showed strong antagonistic activity and consistent reductions in disease parameters. While initial differences among treatments were not statistically significant (p > 0.05), significant effects (p ≤ 0.05 and p ≤ 0.01) emerged over time, indicating a time-dependent impact. Correlation analysis revealed strong relationships among disease incidence, severity, and index, suggesting that these parameters can be used collectively to assess disease progression and treatment efficacy. These findings confirm the promise of selected *P. fluorescens* isolates in sustainable tomato disease management. The more the genotype is treated the more resistance to the fungus and less infected by early blight diseases. As indicated in the use of resistance resources in breeding programs will lead to the production of new cultivars with high performance and resistance to biotic stresses [46–48].

The implications of this research are significant for sustainable agriculture. The use of native microbial antagonists like *P. fluorescens* offers a promising, environmentally friendly approach to managing fungal diseases such as early blight in tomato. Incorporating such biological control agents into integrated pest management (IPM) frameworks could enhance crop protection while minimizing ecological impact and promoting soil health. Moreover, IPM is very crucial not only for controlling tomato but also potato. Types of irrigation play a significant role in the occurrence and development of early blight disease. Furrow irrigation caused higher virulence of early blight disease in comparison with sprinkler and drip irrigation systems. The delay in the planting date can be effective to EB disease management, based on regions condition and cultivars maturity [49].

## Conclusion

This study successfully isolated and characterized the fungal pathogen *Alternaria solani*, the causal agent of tomato early blight, along with several rhizosphere-derived strains of *Pseudomonas fluorescens* collected from rhizosphere soil and infected tomato leaf samples from three major tomato-producing Kebeles, Sirinka, Sanka, and Kobo, located in the North Wollo Zone, Ethiopia. The isolated *Alternaria solani* exhibited significant antagonistic potential. Morphological and microscopic analyses confirmed the identity and diversity of the *A. solani* isolates, while pathogenicity tests validated their virulence on tomato plants. Concurrently, *P. fluorescens* isolates were identified and biochemically characterized, confirming their suitability as biological control agents based on traits such as motility, pigment production, catalase and oxidase activity, and citrate utilization. In vitro dual culture assays demonstrated that all tested *P. fluorescens* isolates suppressed the radial growth of *A. solani*, with isolates Pfs12, Pfk13, and Pfsa31 exhibiting the highest levels of inhibition. These findings were further corroborated by in vivo greenhouse experiments, wherein the same isolates significantly reduced disease incidence, severity, and index compared to untreated controls. Their efficacy was comparable to that of the chemical fungicide mancozeb, indicating their potential as effective bio-control alternatives.

The observed reduction in disease pressure under greenhouse conditions underscores the practical applicability of these *P. fluorescens* strains in integrated disease management strategies. Their ability to suppress *A. solani* without inducing phytotoxic effects suggests they may contribute to reducing reliance on synthetic fungicides, which are often associated with environmental contamination, resistance development, and residual toxicity. Overall, this research demonstrates that selected *P. fluorescens* isolates possess strong potential as bio-control agents against *A. solani*, offering a viable alternative to chemical fungicides and contributing to the development of more sustainable tomato production systems.

## Supporting information

S1 Table (Supporting tables). This file contains S1–S7 in S1 Tables, including analyses of variance for radial growth of the pathogen and treatments under in vitro conditions, percent growth inhibition, percent disease incidence, percent disease severity, percent disease index under greenhouse conditions, and correlation analysis among tomato early blight disease parameters. (DOCX)

S1 Fig (Supporting figures). This file contains S1–S7 in S1 Figs, including biochemical characterization tests of *Pseudomonas fluorescens* and graphical presentations of pathogen radial growth, treatment effects under in vitro conditions, and marginal means of disease incidence, disease severity, and disease index of tomato early blight under greenhouse conditions. (DOCX)

## References

[1] Akram M, et al. Vitamins and minerals: Types, sources and their functions. Functional foods and nutraceuticals: bioactive components, formulations and innovations. 2020. p. 149–72.

[2] Singh and Surender. Agrometeorological requirements for sustainable vegetable crops production. J Food Prot. 2018;2(3):1–22.

[3] Gemechu GE, Beyene TM. Evaluation of tomato (Solanum lycopersicum L. mill) varieties for yield and fruit quality in Ethiopia. A review. Evaluation. 2019;89(10).

[4] Yigezu Wendimu G. The challenges and prospects of Ethiopian agriculture. Cogent Food & Agriculture. 2021;7(1). doi: 10.1080/23311932.2021.1923619

[5] Alizadeh-Moghaddam G, Nasr-Esfahani M, Rezayatmand Z, Khozaei M. Genomic markers analysis associated with resistance to Alternaria alternata (fr.) keissler-tomato pathotype, Solanum lycopersicum L. Breed Sci. 2022;72(4):285–96. doi: 10.1270/jsbbs.22003 36699824 PMC9868332

[6] Pourarian A, Nasr Esfahani M, Sadravi M. Molecular and pathogenic characterization of Iranian isolates associated with leaf spot disease of potato. Acta fytotechn zootechn. 2018;21(1):1–5. doi: 10.15414/afz.2018.21.01.01-05

[7] Panno S, Davino S, Caruso AG, Bertacca S, Crnogorac A, Mandić A, et al. A Review of the Most Common and Economically Important Diseases That Undermine the Cultivation of Tomato Crop in the Mediterranean Basin. Agronomy. 2021;11(11):2188. doi: 10.3390/agronomy11112188

[8] Gatahi DM. Challenges and opportunities in tomato production chain and sustainable standards. International Journal of Horticultural Science and Technology. 2020;7(3):235–62.

[9] Boro M, Sannyasi S, Chettri D, Verma AK. Microorganisms in biological control strategies to manage microbial plant pathogens: a review. Arch Microbiol. 2022;204(11):666. doi: 10.1007/s00203-022-03279-w 36214917

[10] Lahlali R, Ezrari S, Radouane N, Kenfaoui J, Esmaeel Q, El Hamss H, et al. Biological Control of Plant Pathogens: A Global Perspective. Microorganisms. 2022;10(3):596. doi: 10.3390/microorganisms10030596 35336171 PMC8951280

[11] Basit A, Shah ST, Muntha ST, Mohamed HI. Plant Growth-Promoting Rhizobacteria (PGPR) as Biocontrol Agents for Viral Protection. Plant Growth-Promoting Microbes for Sustainable Biotic and Abiotic Stress Management. Springer International Publishing. 2021. p. 187–225. doi: 10.1007/978-3-030-66587-6_8

[12] Kumar Maurya M, Singh R, Tomer A. In vitro evaluation of antagonistic activity of Pseudomonas fluorescensagainst fungal pathogen. JBiopest. 2014;07(01):43–6. doi: 10.57182/jbiopestic.7.1.43-46

[13] M Alhussa K. Morphological and physiological characterization of alternaria solani Isolated from Tomato in Jordan Valley. Research J of Biological Sciences. 2012;7(8):316–9. doi: 10.3923/rjbsci.2012.316.319

[14] Roy CK, Akter N, Sarkar MKI, Pk MU, Begum N, Zenat EA, et al. Control of early blight of tomato caused by alternaria solani and screening of tomato varieties against the pathogen. TOMICROJ. 2019;13(1):41–50. doi: 10.2174/1874285801913010041

[15] Stammler A, et al. Pathogenicity of Alternaria-species on potatoes and tomatoes. 2014.

[16] Hammami I, Ben Hsouna A, Hamdi N, Gdoura R, Triki MA. Isolation and characterization of rhizosphere bacteria for the biocontrol of the damping-off disease of tomatoes in Tunisia. C R Biol. 2013;336(11–12):557–64. doi: 10.1016/j.crvi.2013.10.006 24296079

[17] Kipgen TL, Bora LC, Goswami G, Barooah M, Borah PK, Puzari KC. Isolation and characterization of fluorescent Pseudomonas with bio-control potential against Ralstonia solanacearum. Indian Phytopathology. 2021;74(4):1055–64. doi: 10.1007/s42360-021-00400-9

[18] Bagul, et al. Isolation and characterization of Pseudomonas fluorescens isolates from different rhizospheric soils of Latur district of Maharashtra. 2023.

[19] Nepali B, Bhattarai S, Shrestha J. Identification of Pseudomonas fluorescens using different biochemical tests. IJAB. 2018;2(2). doi: 10.20956/ijab.v2i2.5260

[20] Mohammed AF, Oloyede AR, Odeseye AO. Biological control of bacterial wilt of tomato caused by Ralstonia solanacearum using Pseudomonas species isolated from the rhizosphere of tomato plants. Archives of Phytopathology and Plant Protection. 2020;53(1–2):1–16. doi: 10.1080/03235408.2020.1715756

[21] Soesanto, et al. Biochemical characteristic of Pseudomonas fluorescens P60. Journal of Biotechnology and Biodiversity. 2011;2:19–26.

[22] Onchwari RG. Isolation and characterization of rhizosphere bacteria with potential to improve the plant growth of banana plants in Juja, Kenya. Cohes, Jkuat; 2016.

[23] Anitha G, Kumudini BS. Isolation and characterization of fluorescent pseudomonads and their effect on plant growth promotion. J Environ Biol. 2014;35(4):627–34. 25004745

[24] Güler İ, Küçük Ç. Isolation and characterization of Pseudomonas isolates for antagonistic activities. Journal of Applied Biological Sciences. 2010;3:25–30.

[25] Suresh P, Vellasamy S, Almaary KS, Dawoud TM, Elbadawi YB. Fluorescent pseudomonads (FPs) as a potential biocontrol and plant growth promoting agent associated with tomato rhizosphere. Journal of King Saud University - Science. 2021;33(4):101423. doi: 10.1016/j.jksus.2021.101423

[26] Lalhruaitluangi C, et al. In-vitro evaluation of antagonistic activity of native Trichoderma spp. and Pseudomonas fluorescens isolates against Alternaria solani causing early blight of tomato. International Journal of Plant & Soil Science. 2022;34(13):120–7.

[27] Ahmad T, Nie C, Cao C, Xiao Y, Yu X, Liu Y. First record of Alternaria tenuissima causing Aloe barbadensis leaf blight and leaf spot disease in Beijing, China. Crop Protection. 2024;175:106447. doi: 10.1016/j.cropro.2023.106447

[28] Zegeye, et al. Biocontrol activity of Trichoderma viride and Pseudomonas fluorescens against Phytophthora infestans under greenhouse conditions. Journal of Agricultural Technology. 2011;7(6):1589–602.

[29] Meena B, Marimuthu T. Effect of application methods of pseudomonas jbiopest 5(1): 1-6 fluorescens for the late leaf spot of groundnut management. JBiopest. 2011;5(1):14–7. doi: 10.57182/jbiopestic.5.1.14-17

[30] González-Concha LF, Ramírez-Gil JG, Mora-Romero GA, García-Estrada RS, Carrillo-Fasio JA, Tovar-Pedraza JM. Development of a scale for assessment of disease severity and impact of tomato brown rugose fruit virus on tomato yield. Eur J Plant Pathol. 2022;165(3):579–92. doi: 10.1007/s10658-022-02629-0

[31] Wspanialy P, Moussa M. A detection and severity estimation system for generic diseases of tomato greenhouse plants. Computers and Electronics in Agriculture. 2020;178:105701. doi: 10.1016/j.compag.2020.105701

[32] Matumwabirhi, Kulimushi. Effectiveness of Trichoderma spp., Bacillus spp. and Pseudomonas fluorescens in the management of early blight of tomatoes. University of Nairobi; 2020.

[33] Subhani MN, Ali F, Nasir S, Shahid AA. Isolation, characterization and management of alternaria solani causing early blight in tomato through different bacterial strains. PCBMB. 2022;:78–88. doi: 10.56557/pcbmb/2022/v23i31-327806

[34] Gund P. Studies on variability in Alternaria solani causing early blight of tomato. Maharashtra, India: MPKV Rahuri; 2015.

[35] Saleem A, El-Shahir AA. Morphological and molecular characterization of some alternaria species isolated from tomato fruits concerning mycotoxin production and polyketide synthase genes. Plants (Basel). 2022;11(9):1168. doi: 10.3390/plants11091168 35567169 PMC9103205

[36] Sah SK, Singh AK, Singh BK, Barman K, Pal AK. Screening of tomato genotypes for early blight disease resistance in tomato (Solanum lycopersicum L.). IntJCurrMicrobiolAppSci. 2020;9(10):2909–14. doi: 10.20546/ijcmas.2020.910.350

[37] Gerd Stammler FB, Jasmin P, Simone M, Vanessa T, Pathogenicity of Alternaria-species on potatoes and tomatoes. Fourteenth Euroblight Workshop; 2014.

[38] Kumar et al. Forest ecosystem soil attributes influence density of Pseudomonas fluorescens. Indian Journal of Ecology. 2023;50(3):778–84.

[39] Rahman KHAMK, Yaseen HS. Isolation of Pseudomonas fluorescens from Erbil soils and screening for the presence of siderophores biosynthesis genes. 2024.

[40] Ningaraju T. Collection, isolation and characterization of the Pseudomonas fluorescence, from rhizosphere of different crops (Ragi, pigeonpea and groundnut). IJCS. 2020;8(4):2429–33.

[41] Belkar and Gade. Biochemical characterization and growth promotion activities of Pseudomonas fluorescens. J Pl Dis Sci. 2012;7(2):170–4.

[42] Dugassa A, Alemu T, Woldehawariat Y. In-vitro compatibility assay of indigenous Trichoderma and Pseudomonas species and their antagonistic activities against black root rot disease (Fusarium solani) of faba bean (Vicia faba L.). BMC Microbiol. 2021;21(1):115. doi: 10.1186/s12866-021-02181-7 33865331 PMC8052857

[43] Neidig N, Paul RJ, Scheu S, Jousset A. Secondary metabolites of Pseudomonas fluorescens CHA0 drive complex non-trophic interactions with bacterivorous nematodes. Microb Ecol. 2011;61(4):853–9. doi: 10.1007/s00248-011-9821-z 21360140 PMC3098371

[44] Naglaa, et al. Efficacy of free and formulated arbuscular mycorrhiza, Trichoderma viride and Pseudomonas fluorescens on controlling tomato root rot diseases. Egyptian Journal of Biological Pest Control. 2016;26(3):477.

[45] Kulimushi, et al. Potential of Trichoderma spp., Bacillus subtilis and Pseudomonas fluorescens in the management of early blight in tomato. Biocontrol Science and Technology. 2021;31(9):912–23.

[46] Alizadeh-Moghaddam G, Rezayatmand Z, Esfahani MN-, Khozaei M. Bio-genetic analysis of resistance in tomato to early blight disease, Alternaria alternata. Phytochemistry. 2020;179:112486. doi: 10.1016/j.phytochem.2020.112486 32828067

[47] Schianchi N, Moro G, Serra S, Prota VA. Plant health and food safety. Phytopathologia Mediterranea. 2022;61(1):3–9.

[48] Moghaddam GA, Rezayatmand Z, Nasr Esfahani M, Khozaei M. Genetic defense analysis of tomatoes in response to early blight disease, Alternaria alternata. Plant Physiol Biochem. 2019;142:500–9. doi: 10.1016/j.plaphy.2019.08.011 31445475

[49] Nasr-Esfahani M. An IPM plan for early blight disease of potato Alternaria solani sorauer and A. alternata (Fries.) Keissler. Archives of Phytopathology and Plant Protection. 2020;55(7):785–96. doi: 10.1080/03235408.2018.1489600

