# Supplementary material for: Evaluation of Pseudomonas fluorescens for biocontrol of early blight (Alternaria solani) in tomato in North Wollo, Ethiopia
Source: PLoS One. 2026 Jan 23;21(1):e0341442. doi: 10.1371/journal.pone.0341442 (PMC12829864; doi:10.1371/journal.pone.0341442)
Supplement: S1 Table — (DOCX) [file pone.0341442.s001.docx]

Tables S1–S7

S1 Table. Analysis of variance for the radial growth of pathogen in vitro condition

| Source of  Variation | Degree of Freedom | Sum of  Squares | Mean Square | F | Sig. |
| --- | --- | --- | --- | --- | --- |
| Treatments | 11 | 171.969 | 15.634 | 6344.17 | <.001 |
| Replication | 2 | 0.005 | 0.0024 | 0.015 | 0.985 |
| Day / Time | 5 | 231.046 | 46.209 | 18751.92 | <.001 |
| Treatment*Day | 55 | 30.436 | 0.5534 | 224.57 | <.001 |
| Residual /Error | 142 | 0.350 | 0.0025 |  |  |
| Total | 215 | 433.806 |  |  |  |

S2 Table. Analysis of variance for the radial growth of treatment in vitro condition

| Source of  Variation | Degree of Freedom | Sum of  Squares | Mean  Square | F | Sig. |
| --- | --- | --- | --- | --- | --- |
| Treatments | 11 | 171.969 | 15.634 | 904.76 | <.001 |
| Replication | 2 | 0.298 | 0.149 | 0.891 | 0.412 |
| Day/Time | 5 | 328.496 | 65.699 | 3802.20 | <.001 |
| Treatment*Day | 55 | 30.436 | 0.5534 | 32.03 | <.001 |
| Residual / Error | 142 | 2.454 | 0.0173 |  |  |
| Total | 215 | 533.653 |  |  |  |

S3 Table. Analysis of variance for the percent growth inhibition in vitro condition

| Source of  Variation | Degree of Freedom | Sum of  Squares | Mean  Square | F | Sig. |
| --- | --- | --- | --- | --- | --- |
| Treatments | 11 | 49,457.772 | 4,496.161 | 3362.84 | <.001 |
| Replication | 2 | 15.184 | 7.592 | 4.399 | 0.014 |
| Day /Time | 5 | 740.134 | 148.027 | 110.71 | <.001 |
| Treatment*Day | 55 | 150.1 | 2.73 | 2.04 | 0.0004 |
| Residual / Error | 142 | 189.9 | 1.34 |  |  |
| Total | 215 | 50,553.066 |  |  |  |

S4 Table. Analysis of variance for the percent disease incidence at greenhouse

| Source of  Variation | Degree of Freedom | Sum of  Squares | Mean  Square | F | Sig. |
| --- | --- | --- | --- | --- | --- |
| Treatments | 5 | 1,272.62 | 254.524 | 337.01 | <.001 |
| Replication | 2 | 0.37 | 0.184 | 0.080 | 0.923 |
| Day / Time | 5 | 369.44 | 73.887 | 97.83 | <.001 |
| Treatment*Day | 25 | 164.76 | 6.590 | 8.73 | <.001 |
| Residual / Error | 70 | 52.87 | 0.755 |  |  |
| Total | 107 | 1,860.047 |  |  |  |

S5 Table. Analysis of variance for the percent disease severity at greenhouse

| Source of  Variation | Degree of Freedom | Sum of  Squares | Mean  Square | F | Sig. |
| --- | --- | --- | --- | --- | --- |
| Treatments | 5 | 969.96 | 193.991 | 278.18 | <.001 |
| Replication | 2 | 8.69 | 4.343 | 1.933 | 0.150 |
| Day / Time | 5 | 426.76 | 85.352 | 122.39 | <.001 |
| Treatment*Day | 25 | 164.64 | 6.586 | 9.44 | <.001 |
| Residual / Error | 70 | 48.81 | 0.697 |  |  |
| Total | 107 | 1,618.859 |  |  |  |

S6 Table. Analysis of variance for the percent disease index at greenhouse

| Source of  Variation | Degree of Freedom | Sum of  Squares | Mean  Square | F | Sig. |
| --- | --- | --- | --- | --- | --- |
| Treatments | 5 | 17757.6 | 3551.52 | 700.04 | <.001 |
| Replication | 2 | 42.3 | 21.17 | 0.736 | 0.482 |
| Day / Time | 5 | 6400.9 | 1280.17 | 252.34 | <.001 |
| Treatment*Day | 25 | 2376.1 | 95.04 | 18.73 | <.001 |
| Residual / Error | 70 | 355.1 | 5.07 |  |  |
| Total | 107 | 2,6932.037 |  |  |  |

S7 Table. Correlations between tomato early blight disease growth parameters

|  |  | Treatments of Different Isolates | Percent Disease Incidence | Percent Disease Severity | Percent Disease Index |
| --- | --- | --- | --- | --- | --- |
| Treatments of Different Isolates | Pearson Correlation | 1 | .577^**^ | .472^**^ | .536^**^ |
|  | Sig. (2-tailed) |  | .000 | .000 | .000 |
|  | N | 108 | 108 | 108 | 108 |
| Percent Disease Incidence | Pearson Correlation | .577^**^ | 1 | .935^**^ | .985^**^ |
|  | Sig. (2-tailed) | .000 |  | .000 | .000 |
|  | N | 108 | 108 | 108 | 108 |
| Percent Disease Severity | Pearson Correlation | .472^**^ | .935^**^ | 1 | .982^**^ |
|  | Sig. (2-tailed) | .000 | .000 |  | .000 |
|  | N | 108 | 108 | 108 | 108 |
| Percent Disease Index | Pearson Correlation | .536^**^ | .985^**^ | .982^**^ | 1 |
|  | Sig. (2-tailed) | .000 | .000 | .000 |  |
|  | N | 108 | 108 | 108 | 108 |

**. Correlation is significant at the 0.01 level.

*. Correlation is significant at the 0.05 level
