# Supplementary material for: Evaluation of Pseudomonas fluorescens for biocontrol of early blight (Alternaria solani) in tomato in North Wollo, Ethiopia
Source: PLoS One. 2026 Jan 23;21(1):e0341442. doi: 10.1371/journal.pone.0341442 (PMC12829864; doi:10.1371/journal.pone.0341442)
Supplement: S1 Fig — (DOCX) [file pone.0341442.s002.docx]

**Figures S1–S7**





S1 Figs. Different biochemical test of isolated *P.fluorescens*

(A) Catalase test, (B) Oxidase test, (C) Starch hydrolysis test, (D) *Fluorescens* pigmentation test, (E) Citrate utilization test, (F) Gelatin hydrolysis test, (G) Indole test, (H) motility test, (I) TSI test.





S2 Fig. Mean diameter of radial growth of pathogen in the presence of isolated *Pseudomonas fluorescens*





S3 Fig. Analysis of variance for the radial growth of treatment in vitro condition





S4 Fig. Mean diameter of radial growth of treatment in the presence of isolated *Pseudomonas fluorescens*





S5 Fig. Marginal mean of percent disease incidence for early blight of tomato at greenhouse





S6 Fig. Marginal mean of percent disease severity for early blight of tomato at greenhouse





S7 Fig. Marginal mean of percent disease index for early blight of tomato at greenhouse
